# Global resilience and new strategies needed for antimicrobial stewardship during the COVID‐19 pandemic and beyond

**DOI:** 10.1002/jac5.1622

**Published:** 2022-04-17

**Authors:** Debra A. Goff, Timothy P. Gauthier, Bradley J. Langford, Pavel Prusakov, Michael Ubaka Chukwuemka, Benedict C. Nwomeh, Khalid A. Yunis, Therese Saad, Dena van den Bergh, Maria Virginia Villegas, Nela Martinez, Andrew Morris, Diane Ashiru‐Oredope, Philip Howard, Pablo J. Sanchez

**Affiliations:** ^1^ The Ohio State University Wexner Medical Center, The Ohio State University College of Pharmacy Columbus Ohio USA; ^2^ Baptist Health South Florida Clinical Pharmacy Enterprise Miami Florida USA; ^3^ Public Health Ontario Toronto Ontario Canada; ^4^ Nationwide Children's Hospital Department of Pharmacy Columbus Ohio USA; ^5^ Department of Clinical Pharmacy and Pharmacy Management University of Nigeria Nsukka Enugu Nigeria; ^6^ Department of Pediatric Surgery Nationwide Children's Hospital Columbus Ohio USA; ^7^ Department of Pediatrics, Division of Neonatology American University of Beirut Beirut Lebanon; ^8^ Pharmacy Department American University of Beirut Beirut Lebanon; ^9^ Division of Infectious Diseases & HIV Medicine, Department of Medicine University of Cape Town Cape Town South Africa; ^10^ Grupo de Resistencia Antimicrobiana y Epidemiología Hospitalaria Universidad El Bosque Bogotá Colombia; ^11^ Clinica Imbanaco Cali Colombia; ^12^ Sinai Health System‐University Health Network Toronto Toronto Ontario Canada; ^13^ UK Health Security Agency London UK; ^14^ Commonwealth Pharmacists Association London UK; ^15^ NHS England and NHS Improvement Quarry House Leeds UK; ^16^ Department of Pediatrics, Divisions of Neonatology and Pediatric Infectious Diseases Nationwide Children's Hospital, Abigail Wexner Research Institute at Nationwide Children's Hospital, Center for Perinatal Research Columbus Ohio USA

**Keywords:** antimicrobial stewardship, COVID‐19 pandemic, pharmacists, physicians, SARS‐CoV‐2

## Abstract

Resilience is having the ability to respond to adversity proactively and resourcefully. The coronavirus disease 2019 (COVID‐19) pandemic's profound impact on antimicrobial stewardship programs (ASP) requires clinicians to call on their own resilience to manage the demands of the pandemic and the disruption of ASP activities. This article provides examples of ASP resilience from pharmacists and physicians from seven countries with different resources and approaches to ASP—The United States, The United Kingdom, Canada, Nigeria, Lebanon, South Africa, and Colombia. The lessons learned pertain to providing ASP clinical services in the context of a global pandemic, developing new ASP paradigms in the face of COVID‐19, leveraging technology to extend the reach of ASP, and conducting international collaborative ASP research remotely. This article serves as an example of how resilience and global collaboration is sustaining our ASPs by sharing new “how to” do antimicrobial stewardship practices during the COVID‐19 pandemic.

## 1 INTRODUCTION

In February 2020, global antimicrobial stewardship experts were to attend the World Health Organization (WHO) meeting in Bangkok, Thailand to launch the “Antimicrobial Stewardship Programmes in Health‐care Facilities in Low‐and Middle‐Income Countries: A WHO Practical Toolkit.” However, the coronavirus 2019 (covid‐19) global pandemic postponed the meeting and 2 years later it has yet to be rescheduled. if covid‐19 is the dress rehearsal for the looming antimicrobial resistance (AMR) pandemic, then we know the world is woefully unprepared.

The negative global impact of COVID‐19 on AMR, antimicrobial stewardship programs (ASP), and antimicrobial stewards is emerging. In the United States, health care‐associated infections increased 47% in 2020 compared with 2019.^1^ Multiple countries in the Americas are reporting surges in detection of drug‐resistant infections.^2^ A meta‐analysis of 154 international studies showed that 64% of COVID‐19 patients received antibiotics while the rate of proven bacterial infections was only around 8.6%.^3^ Substantial and persistent disruptions to childhood vaccinations have occurred.^4^ ASP pharmacists from nine high‐income countries (HIC) and low‐middle income countries (LMIC) reported their resources were diverted to support labor‐intensive commitments related to COVID‐19.^5^ Efforts to mitigate these problems require resilience to resourcefully re‐engage ASP initiatives.

The 2022 Global Research on Antimicrobial Resistance (GRAM) report estimates 4.95 million deaths associated with bacterial AMR in 2019, including at least 1.27 million deaths attributable to bacterial AMR.^6^ This analysis found that the highest mortality burden from AMR was in LMIC. The findings highlight the need for urgent, accelerated action in ASP in both LMIC and HIC. This article aims to provide examples and personal testimonies of local, national, and international ASP resilience from antimicrobial stewards in seven countries with different resources and approaches to ASP. We describe our ASPs before COVID‐19 and share our new strategies during the COVID‐19 pandemic to keep moving forward during these difficult times.

## 2 METHODS

An open‐call was made via email in October 2021 to ASP pharmacists and physicians in HIC and LMIC to describe examples of their ASP resiliency during the pandemic that could help colleagues in other parts of the world. Sharing creative solutions can inform all stewards how to carry on with competing pressures from the additional COVID‐19 workload and stress.

## 3 RESULTS

Contributions were received from seven countries—The United States, The United Kingdom (UK), Canada, Nigeria, Lebanon, South Africa (SA), and Colombia who describe the ASP challenges and mitigation strategies in their local settings (Table 1).

**TABLE 1:** Examples of resilience by antimicrobial stewardship programs during the COVID‐19 pandemic

Challenge	Mitigation strategy	
Antibiotic use	High income country	Low–middle income country
*Supply chain challenges* Antibiotic shortages can drive antibiotic resistance through replacement of broader‐spectrum antibiotics or fake drugs	In the United States, ASP pharmacists developed strategies to deal with antibiotic shortages which involved evaluating and providing therapeutic alternatives	Nigerian pharmacists helped design new responsive supply chain systems for efficient distribution of antimicrobials
		Pharmacists in Colombia were reassigned to review prescription trends to secure antimicrobials which had a shortage
*Inappropriate antibiotic use* Risk of antibiotic overuse as speculative therapy for COVID‐19 and due to diagnostic uncertainty and/or concern for bacterial co‐infection	Canada's interdisciplinary working groups developed appropriate empiric antibiotic use recommendations to reduce the unnecessary use of antibiotics. The Choosing Wisely Canada updated its *Cold Standard Toolkit* to address the management of respiratory tract infections in the context of COVID‐19 and virtual care^7^	Colombian infectious diseases physicians developed diagnostic and treatment protocols for COVID‐19 with pharmacists in charge of following those protocols, as well as monitoring for drug interactions
	US pharmacists and physicians contributed to COVID‐19 local guidelines and real‐time guidelines by the IDSA^8^ and the NIH.^9^ The CDC hosted weekly multidisciplinary Zoom meetings to allow for real‐time interactive sharing of COVID‐19 best practices	
Infection control/prevention		
*Shortages of hand sanitizer*	UK pharmacy teams learned how to locally produce WHO‐formula alcohol hand rub from colleagues in Zambia, and were able to locally produce alcohol hand rubs in their hospitals when the United Kingdom became a resource‐limited setting for alcohol hand rub at the start of the COVID‐19 pandemic	Pharmacists in SA and Nigeria augmented the local production of high‐quality hand sanitizers in the face of illegal large‐scale production of sub‐standard products by unapproved vendors^5^
*COVID‐19 vaccine roll‐out*	ASP from the United States, United Kingdom, and Canada assisted in COVID‐19 vaccination programs	
Health care infrastructure		
*Evolving roles and new COVID‐19 responsibilities*	In the United Kingdom, the pandemic broke down silo working and barriers, resulting in increased multi‐disciplinary and cross‐sector collaborations	The pandemic energized the Nigeria Center for Disease Control to develop and test ASP policy in select hospitals
*ASPs were tasked with new COVID‐19 responsibilities in addition to their daily work*	In the United States, the ASP at Baptist Health South Florida transformed their ASP electronic reports by overlapping azithromycin prescribing for acute respiratory infections alongside azithromycin prescribing for COVID‐19	Per WHO recommendations and the SA National Department of Health, a pharmacist designed a Monitored Emergency Use of Unregistered Interventions study to ensure the safe and effective monitoring of patients receiving off‐label therapeutics for COVID‐19 outside of a clinical trial
*Maintaining ASP activities*	The United Kingdom, Canada, and the United States used existing technology resources to facilitate antibiotic stewardship meetings and virtual ward rounds	
*Disparities in financial resources to support ASPs expanded responsibilities during the pandemic*		Nigerian hospitals collaborated with high income countries to secure funding from the US Agency for International Development and the UK's Fleming Fund^10^ to increase AMR awareness, strengthen ASPs, and provide support for lab equipment and microbiology personnel training for 18 hospitals Lebanon ASP at AUBMC in collaboration with U.S. partners at NCH and OSU secured funding to implement multidisciplinary ASPs in 6 public hospitals across Lebanon
ASP Research		
*Competing priorities caused ASP research to be deprioritized*, *delayed*, *or halted*	New networks of collaborators formed to efficiently tackle COVID‐19 issues. ASPs in the United States, United Kingdom, and Canada facilitated participation in COVID‐19 drug therapy clinical trials	ASPs in Lebanon and S.A. used videoconferencing as a new way of engaging with participants across geographies with the added benefit of recording sessions that can be watched later for those unable to attend
*Travel restrictions and physical distancing halted sharing of information at conferences*, *in‐person meetings*, *and networking activities*	A neonatal ASP research study with US and SA was halted in 2020 with much resilience resumed in late 2021	The WHO published measures to ensure that antimicrobial stewardship is integrated into pandemic responses^11^
	OSU global ASP used videoconferencing to network and engage with partners in SA, Lebanon, and Latin America NCH faculty used videoconferences to teach SA faculty how to use the online database Redcap for a research study	A new international ASP initiative across Latin America including Argentina, Brazil, Peru, Chile, Colombia, Costa Rica, and Mexico in collaboration with US partners at OSU is focusing on the role of pharmacists in ASP interventions. A virtual ASP training course was developed with interventions and outcomes

Abbreviations: AMR, antimicrobial resistance; ASP, antimicrobial stewardship programs; AUBMC, American University of Beirut Medical Center; CDC, Centers for Disease Control and Prevention; COVID‐19, coronavirus disease 2019; IDSA, Infectious Diseases Society of America; NCH, Nationwide Children's Hospital; NIH, National Institute of Health; OSU, The Ohio State University; SA, South Africa; UK, United Kingdom; US, United States; WHO, World Health Organization.

## 4 ANTIMICROBIAL STEWARDSHIP IN LOW–MIDDLE INCOME COUNTRIES PRIOR TO THE PANDEMIC

### 4.1 Nigeria

ASPs are sub‐optimal in Nigeria and this contributes to AMR.^12^ A 2019 survey on the status of ASP in Nigeria revealed only 24% of tertiary hospitals have an ASP, only 6% monitor antimicrobial use, and only two had antibiograms.^13^ The most common barriers to pharmacists' involvement in ASP were lack of education and training in ASP and infectious diseases.^14^ Nigerian physicians recognize AMR as a problem but only 28% had heard of ASPs.^12^ Hospitals routinely experience antibiotic shortages and this was exacerbated during the pandemic. Physicians often must instruct their patients to purchase antibiotics from local pharmacies and bring the supply to the hospital. Tracking accurate hospital antibiotic use in this scenario is almost impossible. COVID‐19 has diverted much attention away from ASP in the Nigerian National Action Plan for AMR.

### 4.2 Lebanon

After starting ASP efforts in 2017, the American University of Beirut Medical Center (AUBMC) launched an ASP in 2018 with a dedicated physician and pharmacist. The national AMR committee published in 2019 a national action plan in line with the WHO global action plan that included the activities to address AMR.^15^ Some private academic institutions designed successful ASPs including one that reduced neonatal antibiotic usage by 63%,^16^, ^17^ however most public hospitals struggle to implement ASP.

### 4.3 Colombia

Many hospitals are in the process of developing ASPs while some have established ASPs. Pharmacists in Colombia generally do not have dedicated ASP time and focus mostly on dispensing medicines. The most recent national strategy published in 2018 is the “Program for the Prevention, Surveillance and Control of Hospital Acquired Infections (HAI) and Antimicrobial Resistance,”^18^ which aims to reduce the occurrence of HAI and their consequences, promoted by law for the implementation of ASP in all Colombian hospitals.

### 4.4 South Africa

The S.A. multidisciplinary ASP was formed in 2015. Despite significant constraints, SA physicians, pharmacists, and microbiologists successfully used existing resources to implement ASP with pharmacist interventions that decreased antibiotic use by 18%.^19^ One study with 69 pharmacists from 39 public and private hospitals collaborated to successfully implement stewardship interventions and improve compliance with national community‐acquired pneumonia guidelines.^20^ The launch of the SA National Neonatal Sepsis Task Force^21^ in 2019 activated a new national study in collaboration with U.S. ASP experts from Nationwide Children's Hospital (NCH) and The Ohio State University (OSU) to implement neonatal ASP in SA hospitals to address the high neonatal mortality associated with AMR.

## 5 ANTIMICROBIAL STEWARDSHIP IN HIGH INCOME COUNTRIES PRIOR TO THE PANDEMIC

### 5.1 United States

ASPs are required in all US acute care hospitals. In 2018, 85% of acute care hospitals report having all seven of the Centers for Disease Control and Prevention (CDC) ASP Core Elements in place.^22^ Regulatory requirements for outpatient ASP went into effect in January 2020. One major focus is to address inappropriate prescribing of antibiotics for acute respiratory infections (ARI). At Baptist Health South Florida, a system including over 50 outpatient centers, an ARI Campaign was an outpatient stewardship goal. Elsewhere at OSU, global outreach and collaboration to combat AMR was a 2020 goal. The OSU program director for the *Train the Trainer* ASP for SA pharmacists^23^ in collaboration with the Society of Infectious Diseases Pharmacists (SIDP) were to start work with the WHO to help implement the training of pharmacists from LMICs for participation in ASPs. However, the pandemic disrupted both initiatives.

### 5.2 Canada

ASPs are an accreditation requirement in Canadian hospitals,^24^ but there is more work to be done in all Canadian practice settings. ASP efforts have continued to expand into the long‐term care (eg, focus on reducing testing and treatment for asymptomatic bacteriuria^25^) and primary care setting (eg, focus on reducing unnecessary antibiotic use for upper respiratory tract infections^26^).

### 5.3 United Kingdom

There are national requirements for AMS built into all health care provider contracts with leadership at a national level and organizational delivery against contractual requirements and performance monitoring. In addition, England recently appointed seven posts to lead on regional (population: 8 million) quality improvement and performance delivery that tailors local needs to national drivers.^27^ Improvements to open access data on prescribing and AMR also support identifying opportunities remotely for ASP improvement.^28^

## 6 RESILIENCY OF ASPs DURING THE COVID‐19 PANDEMIC

The demands of the pandemic are unlike any previous ASP responses to outbreaks or the influenza season. There was no “COVID‐19 playbook” to refer to. Developing COVID‐19 drug therapy guidelines and providing multiple revisions diverted ASPs from daily audit and feedback interventions. While we would like to assume that these added responsibilities by ASPs strengthened the value and funding of ASPs, a recent study reports otherwise. The WHO Global Antimicrobial Resistance and Surveillance System (GLASS), conducted a survey in 73 countries to assess the effects of COVID‐19 on AMR activities.^29^ Over 67% of LMIC reported decreased funding for AMR and ASPs are working with fewer resources during the pandemic. Table 1 describes examples of resiliency demonstrated by ASPs in seven countries.

### 6.1 Nigeria

New local efforts by pharmacists, physicians, and microbiologists at the University of Nigeria Teaching Hospital are addressing ASP practices amidst increased utilization of antimicrobials by the public during the pandemic.^30^ Nigeria is particularly vulnerable to disruptions in global supply chains because about 70% of the drug supply is imported.^31^ During the pandemic, pharmacists play an integral role in securing medications during drug shortages.^32^

### 6.2 Lebanon

At the start of the pandemic, all ASP activities at AUBMC fixated on mitigation strategies to control COVID‐19. Simultaneously, Lebanon was struggling to overcome unprecedented economic and financial crisis and political unrest, in addition to a massive blast in August 2020 that destroyed half of Beirut, damaged major hospitals, and left more than half a million citizens injured physically, economically, and psychologically.^33^, ^34^ The health care sector is on the verge of a complete collapse due to the increase in population poverty and massive shortages in the workforce, diagnostics, medical supplies, and medications, increasing the risk for AMR.^35^ Because the public resilience response is focused on strengthening the primary health care network to build long‐term social solidarity,^35^ the ASP at AUBMC is expanding ASPs to the national level using the *Train the Trainer* pharmacist model.^36^ While challenges have occurred with this ASP project within the current national crisis and the dire economic situation, the health care providers at the six participating hospitals show resilience and determination to implement ASPs.

### 6.3 South Africa

SA ASP was faced with the diversion of health care resources to the COVID‐19 response. The international neonatal intensive care unit (NICU)‐ASP research collaboration formed in early 2020 between US partners at OSU‐NCH and 15 hospitals across SA faced inevitable delays and a threat of abandonment. However, the study faculty comprising over 20 multidisciplinary professionals leveraged their individual and collective resilience to adapt the approach and enable the study to continue. Importantly, the international collaboration has provided a professional network that has sustained motivation and support for ASP teams

### 6.4 Colombia

During the pandemic, most ASP activities in Colombia were stopped or limited given the need to support COVID‐19 patients. Roles were reassigned, and new ASP strategies include virtual teleconferencing for education on new protocols and therapies. Resilience enabled the development and implementation of a new international ASP collaboration between hospitals in Columbia, Argentina, Chile, Brazil, Costa Rica, Mexico, Peru, and the United States.

### 6.5 Canada

Although COVID‐19 diverted resources and attention away from AMR and ASP in Canada, including the development and release of a National Action Plan on AMR as part of the COVID‐19 response, new frameworks and connections have been established that can help to address AMR moving forward. In several of Canada's provinces, interdisciplinary clinical practice guideline working groups were formed to develop evidence‐based recommendations to improve the safe, effective, and equitable use of COVID‐19 pharmacotherapy.^37^ Given the concern for bacterial co‐infection in patients with COVID‐19, appropriate empiric antibiotic use recommendations were also developed to reduce the unnecessary use of antibiotics in these patients.^37^

The Canadian Institutes for Health Research provided funding for COVID research, including the support of randomized clinical trials such as SOLIDARITY.^38^ The existing infrastructure and new relationships created will form a solid basis for future clinical trials that optimize the management of COVID‐19. An interdisciplinary team of pharmacists, physicians, and trainees initiated a living review and meta‐analysis to describe the prevalence of bacterial co‐infections and antibiotic use in patients with COVID‐19 across the globe and which was updated as the pandemic progressed.^3^, ^39^, ^40^ These findings helped to inform local and international guidelines on the appropriate use of antibiotics in COVID‐19 and led to a collaboration underway between Canadian researchers and the WHO to evaluate AMR in COVID‐19 patients.

### 6.6 United Kingdom

A UK‐wide survey showed that COVID‐19 had a significant negative impact on local ASP activities that included auditing antimicrobials, quality improvement initiatives, education, ASP meetings, and multidisciplinary ward rounds.^41^ One UK pharmacy survey classified respondents as high‐risk within the burnout scale and several pharmacists stated “I'm at a breaking point.”^42^ Resilience and persistence led to new innovation during COVID‐19 that broadly falls into four main categories: (i) digitization, (ii) workforce changes, (iii) service integration, and (iv) person‐centered care.^43^

Although competing priorities to deal with the pandemic have led to reduced focus on global strategies to tackle ASP, there have been pockets of continued focus and innovative ASP practices with UK infection leads supporting and collaborating with colleagues in LMICs through the International Federation of Pharmacists (FIP) and the Commonwealth Partnerships for Antimicrobial Stewardship (CwPAMS).

### 6.7 United States

In a survey of ASP leaders at 51 US hospitals, stewards reported additional duties related to COVID‐19, fewer resources, more work hours, decreased ability to carry out ASP initiatives, and more symptoms of burnout than before the pandemic.^44^ One ASP spent over 300 hours on 85 updates of COVID‐19 guidelines in 2020.^45^ Patient care rounds became a hybrid of virtual and in‐person. ASP pharmacists continued to review patients' charts virtually via remote access to electronic medical record and made recommendations via secure chat function and via phone calls for urgent issues.

International established partnership between NCH, OSU, and SA and on‐going ASP research came to a halt at the start of the pandemic and continues to have delays with every COVID‐19 surge. Perseverance and a large amount of resilience from ASP thought leaders in the United States and LMIC is required to keep our ASP studies going. Sharing Antimicrobial Reports for Paediatric Stewardship (SHARPS),^46^ utilized webinars to share COVID‐19 related experiences among more than 70 pediatric hospitals across the United States, Canada, and the United Kingdom. In addressing what is to come, overlapping new COVID‐19 tools with existing ASP tools is likely to enable resiliency in ASP.

## 7 NEW PARADIGMS IN ANTIMICROBIAL STEWARDSHIP PROGRAMS

**FIGURE 1:**
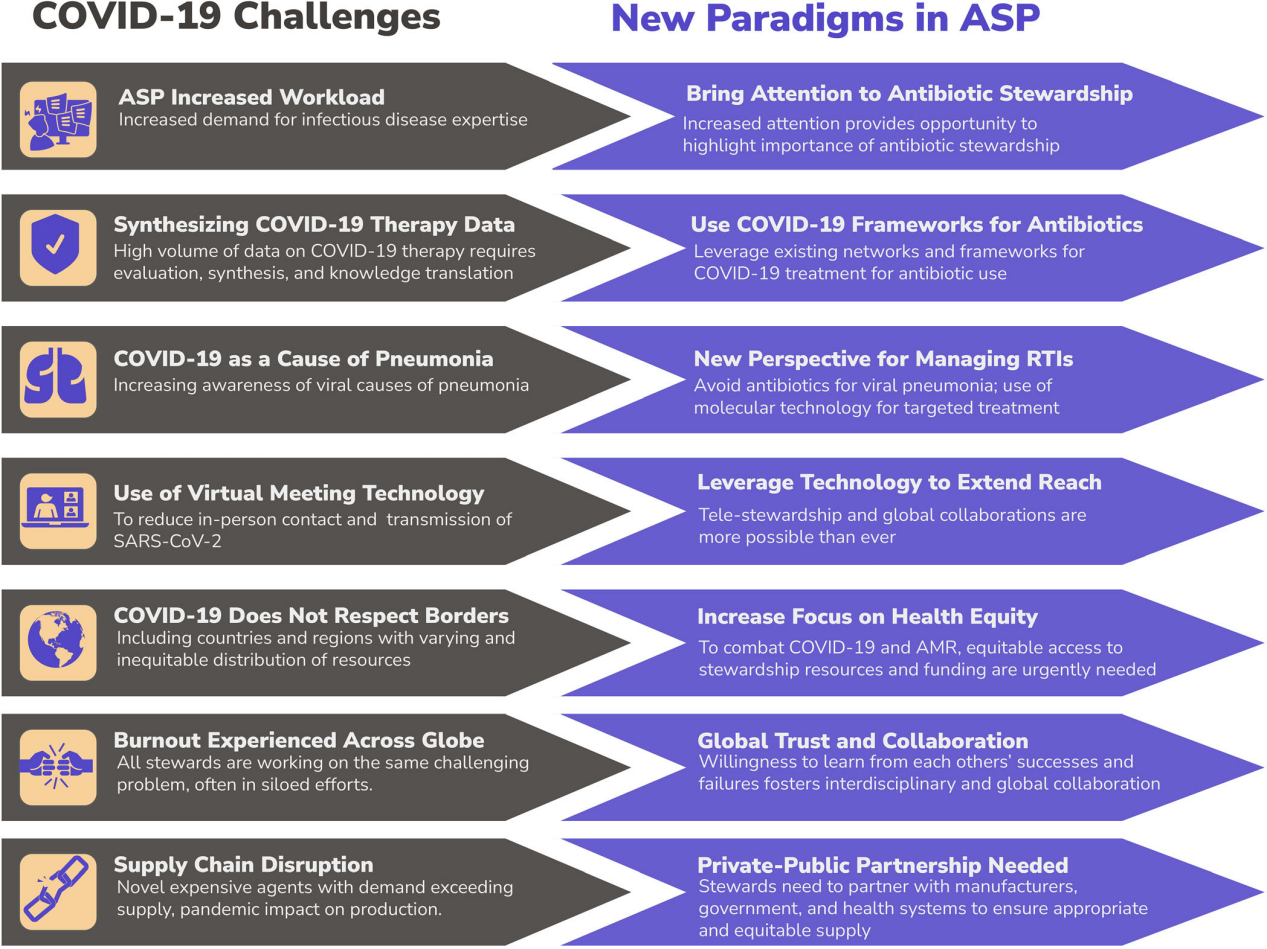
New paradigms in antimicrobial stewardship during COVID‐19. ASP antimicrobial stewardship programs

Figure 1 depicts new paradigms in ASP as a result of the COVID‐19 challenges that have been experienced. ASP pharmacists and physicians rapidly developed and continually updated COVID‐19 guidelines, highlighting the importance and adaptability of ASPs. They have learned to leverage technology by implementing telestewardship, and hosting meetings virtually via Zoom. To combat COVID‐19 and AMR, ASP pharmacists and physicians must assure equitable access to antimicrobials and stewardship resources.^47^ They found new ways to continue international‐focused research to address uncontrolled use of antimicrobials and AMR. While research funding on COVID‐19 has progressed during the pandemic, prioritized funds at the national and international level are needed to prevent the next looming pandemic of AMR.^48^

## 8 GLASS HALF EMPTY OR HALF FULL APPROACH TO ASP DURING COVID‐19

In 2016, the United Nations called on countries to commit to the development and implementation of national AMR action plans. In response, several authors of this paper co‐authored a Personal View that described how ASPs from five HIC and LMIC countries collaborated to answer the UN call to action.^49^ Five years later, in many countries, the plans exist only on paper. The 2021 Tripartite (WHO, World Organisation for Animal Health, and Food and Agriculture Organization) AMR survey revealed that of the 163 responding countries, over 90% reported that the pandemic impeded the development, implementation, and funding of national plans to address AMR. Only 50% have a functional mechanism to prioritize, cost, implement, and monitor AMR national action plans.^50^ The survey highlights the urgent need to build capacity to ensure the effective functioning of ASPs worldwide. In addition, there is a new WHO “Call to Action on AMR—2021” to raise global ambition on AMR.^51^ It calls for enhanced national and global efforts to tackle AMR, for the acceleration of previous commitments to tackle AMR, and for improved ASP. As of 20 September 2021, there are 113 Member State signatories to the Call to Action. While the intent is good, will it be followed by meaningful action? Without ASP funding from the governments responsible for executing the national action plans, the implementation of effective ASP interventions is left to enthusiastic individuals or groups who look at the glass as half full.

As the world enters the third calendar year of the pandemic, there is deep concern for the burnout rate of pharmacists and physicians co‐leading ASPs, waning political will for AMR, and appallingly low levels of ASP funding. Antimicrobial stewards have never encountered a workload and stress this intense for months on end with every COVID surge and new variant. The ASP global workforce needs each other to build resilience to cope with the demands of the pandemic. The resilient efforts of seven ASPs from HIC and LMIC to sustain ASPs to keep moving forward are highlighted. Personal relationships, individual efforts, and willingness to learn from other's successes and failures during the pandemic continues to foster collaboration. As society demonstrates intolerance to the longevity of the pandemic, this does not bode well for ASP messaging to engage the public in the fight against AMR, the “silent pandemic.” Continued disruptions in ASP will fuel the expansion of AMR globally. It will take a deep reservoir of resilience by ASP pharmacists, physicians, microbiologists, infection preventionists, and nurses to keep the focus on AMR and keep ASPs moving forward.

## CONFLICT OF INTEREST

Debra A. Goff; Timothy P. Gauthier; Bradley J. Langford; Pavel Prusakov; Ubaka Chukwuemka M; Benedict C. Nwomeh; Khalid A. Yunis; Therese Saad; Maria Virginia Villegas; Diane Ashiru‐Oredope and Pablo J. Sanchez: see conflict of interest disclosure form submitted with manuscript. Andrew Morris, Philip Howard, Dena van den Bergh, and Nela Martinez have no relevant conflicts of interest.
